# Overexpression of MALT1-A20-NF-κB in adult B-cell acute lymphoblastic leukemia

**DOI:** 10.1186/s12935-015-0222-0

**Published:** 2015-07-25

**Authors:** Yi Xu, Junyan Hu, Xu Wang, Li Xuan, Jing Lai, Ling Xu, Shaohua Chen, Lijian Yang, Gengxin Luo, Kanger Zhu, Xiuli Wu, Yangqiu Li

**Affiliations:** Institute of Hematology, Jinan University, Guangzhou, 510632 China; Department of Emergency, Third Affiliated Hospital, Guangzhou Medical University, Guangzhou, 510150 China; Key Laboratory for Regenerative Medicine of Ministry of Education, Jinan University, Guangzhou, 510632 China; Department of Hematology, Nanfang Hospital, Southern Medical University, Guangzhou, 510515 China; Department of Hematology, First Affiliated Hospital, Jinan University, Guangzhou, 510632 China

**Keywords:** B-cell acute lymphoblastic leukemia, A20, MALT1, NF-κB

## Abstract

**Background:**

A20 is a dual inhibitor of NF-κB activation and apoptosis in the tumor necrosis factor receptor 1 signaling pathway, and both are related to tumorigenesis. A20 is frequently inactivated by deletions and/or mutations in several B and T cell lymphoma subtypes; however, knowledge of the role of A20 in B-cell acute lymphoblastic leukemia (B-ALL) remains limited. In this study, we characterized the A20 gene expression pattern, the expression level of its upstream regulating factor MALT1, and its downstream target NF-κB in adult B-ALL.

**Methods:**

The expression level of MALT1, A20 and NF-κB1 was detected in peripheral blood mononuclear cells (PBMCs) from 20 patients with adult B-ALL (including 12 de novo B-ALL and 8 refractory/relapse B-ALL cases), and nine patients with B-ALL in complete remission (CR) using real-time PCR. Sixteen healthy individuals served as controls.

**Results:**

Significant A20 overexpression was found in the B-ALL (median: 13.489) compared with B-ALL CR (median: 3.755) (*P* = 0.003) patients and healthy individuals (median: 8.748) (*P* = 0.002), while there was no significant difference in A20 expression between B-ALL CR patients and healthy individuals (*P* = 0.107). Interestingly, the A20 expression level in the B-ALL samples was relatively different with approximately 50% of the B-ALL cases showing a relatively high *A20* expression level, while the remaining 50% cases demonstrated slight upregulation or a similar expression level as the healthy controls. However, there was no significant difference in the A20 expression level between de novo B-ALL (median 12.252) and refractory/relapse B-ALL patients (median 21.342) (*P* = 0.616). Similarly, a significantly higher expression level of NF-κB1 was found in the B-ALL (median 1.062) patients compared with healthy individuals (median 0.335) (*P* < 0.0001), while the NF-κB1 expression level was downregulated in the B-ALL CR group (median 0.339), which was significantly lower than that in those with B-ALL (*P* = 0.001). Moreover, the MALT1 expression level in B-ALL was upregulated (median 1.938) and significantly higher than that in healthy individuals (median 0.677) (*P* = 0.002) and B-ALL CR patients (median 0.153) (*P* = 0.008). The correlation of the expression levels of all three genes was lost in B-ALL.

**Conclusions:**

We found that MALT1-A20-NF-κB is overexpressed in adult B-ALL, which may be related to the pathogenesis of B-ALL, and this pathway may be considered a potentially attractive target for the development of B-ALL therapeutics.

**Electronic supplementary material:**

The online version of this article (doi:10.1186/s12935-015-0222-0) contains supplementary material, which is available to authorized users.

## Background

Adult B-cell acute lymphoblastic leukemia (B-ALL) results from clonal malignant B-cell proliferation arising from genetic alterations that block lymphoid differentiation and drive aberrant cell proliferation and survival. B-ALL is the most common hematologic malignancy in children. The cure rate for B-ALL in children is 80%, while it is less than half in adults. Some genetic markers for B-ALL have been found to have prognostic impact [1]. Although great strides have been made toward improving B-ALL outcome, the prognosis for disease relapse has significantly lagged behind, particularly for adults with B-ALL. Understanding the underlying genomic lesions and epigenetic regulatory mechanisms associated with leukemic transformation, drug resistance and disease relapse is essential to revolutionizing leukemia care in this era of personalized medicine. Recently, advances in cytogenetics utilizing array-based technologies and next-generation sequencing (NGS) have revealed exciting insights into the genetic basis of B-ALL [2–6].

A20 (also known as TNFAIP3) is a cytoplasmic protein that was first identified as a tumor necrosis factor (TNF) primary response transcript encoding a 790 amino acid protein with a unique zinc finger motif [7]. The C-terminal domain of A20 (residues 427–790) is critical for its modulation of NF-κB, while its N-terminal deubiquitinase domain may promote the de-ubiquitination of K63-polyubiquitin chains. A number of studies have indicated that A20 is a potent anti-inflammatory signaling molecule that restricts multiple intracellular signaling cascades and the importance of A20-mediated regulation of ubiquitin-dependent signaling in autoimmune diseases and cancer [8, 9].

Thus, A20 play crucial physiological roles as a dual inhibitor of NF-κB activation and apoptosis in the tumor necrosis factor (TNF) receptor 1 signaling pathway. A20 inactivation results in constitutive NF-κB activation, and it is linked to autoimmune and malignant disease, particularly for B-cell lymphomas. In B-cell lymphoma, A20 is thought to act as a tumor suppressor because constitutive NF-κB activation has been implicated in tumorigenesis. As a negative regulator of NF-κB, A20 plays a pivotal role in the regulation of the immune response and prevents excessive NF-κB activation in response to a variety of external stimuli. Recent genetic studies have shown that A20 is a common genetic target in B-lineage lymphomas. A20 is frequently inactivated by somatic mutations and/or deletions in different subtypes of B-cell non-Hodgkin lymphomas, Hodgkin’s lymphoma, and T-cell malignancy-Sézary syndrome (SS). Moreover, A20 promoter methylation is also frequently detected in these lymphomas. When wild-type A20 was re-expressed in a lymphoma-derived cell line, there was cell growth suppression and the induction of apoptosis accompanied by the downregulation of NF-κB activation, while down-regulation of A20 expression by siRNA in Epstein-Barr virus-infected lymphoblastoid cell lines was associated with apoptosis resistance and enhanced clonogenicity. Overall, the uncontrolled NF-κB signaling caused by a loss in A20 function is involved in the pathogenesis of subsets of B-lineage lymphomas [10–14].

In contrast, A20 was initially identified as a primary gene product following TNFα treatment in human umbilical vein endothelial cells, and it has been shown to inhibit TNF-induced apoptosis. It was also shown that A20 has an anti-apoptotic function via the suppression of c-jun N-terminal kinase (JNK) by targeting apoptosis signal-regulating kinase1 (ASK1) [15]. A20 has been found to have antiapoptotic function in several cancer cells, its increased expression is associated with tumorigenesis, and it is involved in drug resistance in many cancers, such as glioblastomas, breast cancer, hepatocellular carcinoma, and nasopharyngeal carcinoma (NPC) [16–21]. For example, A20 has been shown to protect MCF-7 breast carcinoma cells from TNFα-induced apoptosis. In addition, high A20 expression levels were observed in more aggressive breast tumors (ER-negative, progesterone receptor-negative and high histological grade). Furthermore, A20 is a key protein involved in tamoxifen resistance [20]. A20 is overexpressed in glioblastoma stem cells, and inhibiting A20 expression by targeting its mRNA with short hairpin RNAs decreases glioblastoma stem cell growth and survival, and the tumorigenic potential of treated glioblastoma stem cells decreases, resulting in increased survival of mice bearing human glioma xenografts [22]. In this scenario, A20 is thought to be an oncogene.

Inactivating mutations of A20 in lymphoma suggest that A20 can act as a tumor suppressor; however, similar point mutations in A20 may function as tumor enhancers in glioma via the promotion of glioblastoma stem cell survival [22]. Although it is known that A20 has dual functions and both are related to tumorigenesis, why and what function A20 plays in different cancers remain largely unknown. In this study, we characterized the gene expression pattern of A20 and the gene expression level of the upstream regulating factor MALT1 and its target NF-κB in adult B-ALL.

## Methods

### Samples

The samples used in this study were derived from 20 cases with adult B-ALL, including 12 newly diagnosed cases, untreated patients with B-ALL (7 males and 5 females; 17–72 years old, median age: 27.0 years) and eight cases with refractory/relapse B-ALL (3 males and 5 females; 16–59 years old, median age: 30.5 years). The clinical information of the B-ALL cases is listed in Table 1. Nine cases with adult B-ALL in complete remission (CR) (6 males and 3 females; 17–56 years old, median age: 28.0 years) were analyzed in this study. Sixteen healthy individuals including 10 males and 6 females (17–45 years old, median age: 26 years) served as controls. Peripheral blood mononuclear cells (PBMCs) were isolated from heparinized venous blood by Ficoll-Paque gradient centrifugation. RNA extraction and cDNA synthesis from PBMCs were performed according to the manufacturer’s instructions. All human peripheral blood samples were obtained with consent. All procedures were conducted according to the guidelines of the Medical Ethics Committee of the Health Bureau of Guangdong Province in China, and ethical approval was obtained from the Ethics Committee of the Medical School of Jinan University.

**Table 1:** The primers of quantitative real-time RT-PCR [23–25]

Primer	Sequence	Accession no.
A20 For	5′-CTGGGACCATGGCACAACTC-3′	NM_006290
A20 Rev	5′-CGGAAGGTTCCATGGGATTC-3′	
MALT1 For	5′-TCTTGGCTGGACAGTTTGTGA-3′	NM_006785.2
MALT1 Rev	5′-GCTCTCTGGGATGTCGCAA-3′	
NF-κB1 For	5′-CCACAAGACAGAAGCTGAAG-3′	NM_003998
NF-κB1 Rev	5′-AGATACTATCTGTAAGTGAACC-3′	
β_2_M For	5′-TACACTGAATTCACCCCCAC-3′	J00105
β_2_M Rev	5′-CATCCAATCCAAATGCGGCA-3′	

### Quantitative real-time RT-PCR (qRT-PCR)

The primers used in this study for MALT1, A20 and NF-κB1 gene amplification were previously described (Table 1) [23–25]. The expression level of the MALT1, A20, NF-κB1 and β2-microglobulin (β_2_M) genes was determined by SYBR Green I real-time RT-PCR as previously described [23, 24, 26, 27]. The relative amounts of the genes of interest and the β_2_M reference gene were measured in two independent assays. The specific amplified PCR products were analyzed by melting curve analysis. The data are presented as the relative expression of the genes of interest compared with the internal control gene as determined by the 2(^−∆CT^) method [23, 24, 28–31].

### Statistical analysis

Two independent-samples Wilcoxon tests were performed to compare the median expression level of the A20, MALT1 and NF-κB1 genes among patients with B-ALL and B-ALL CR and healthy individuals. Spearman’s correlation and linear regression analyses were used to determine the association between different genes in different groups. A *P* < 0.05 was considered statistically significant [23, 24, 28, 29].

## Results and discussion

**Figure 1:**
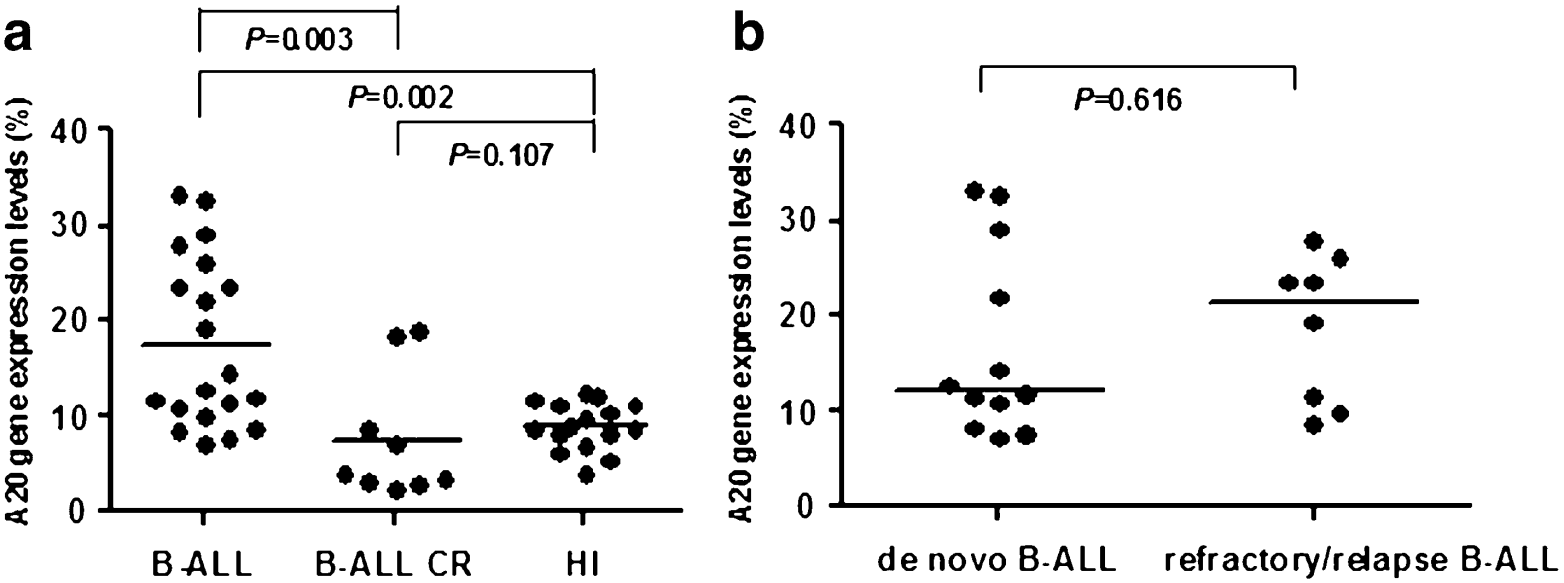
A20 expression in peripheral blood mononuclear cells from patients with B-ALL and healthy individuals. **a** Comparison of the expression level of A20 in the B-ALL, B-ALL CR and healthy individual (HI) groups; **b** comparison of the expression level of A20 in the de novo B-ALL and refractory/relapse B-ALL groups.

A20 is a potent anti-inflammatory signaling molecule that restricts multiple intracellular signalling cascades. A20 deficiency results in hyperactivity and loss of immune homeostasis in B cells. Recent studies have converged to highlight the clinical and biological importance of A20. Polymorphisms and mutations in the A20 gene are linked to various human autoimmune conditions and malignant diseases, and A20 inactivation is a frequent event in human B-cell lymphomas characterized by constitutive NF-κB activity, which is a major hallmark of B-cell malignancies. NF-κB-dependent genes are involved in anti-apoptosis, cell proliferation and metastasis and are responsible for the survival and proliferation of tumors. Moreover, biochemical studies have unveiled complex mechanisms by which A20 regulates ubiquitin-dependent NF-κB and cell-survival signals [8]. Although a number of reports have shown that A20 is frequently inactivated by deletions and/or mutations in several lymphoma subtypes [10–14, 32, 33], little is known about the role of A20 in B-ALL. In this study, we compared the A20 expression level in PBMCs from patients with B-ALL (including de novo and refractory/relapse), patients with B-ALL in complete remission (CR) and healthy individuals. Significant overexpression of A20 was found in B-ALL (median 13.489) compared with B-ALL CR patients (median 3.755) (*P* = 0.003) and healthy individuals (median 8.748) (*P* = 0.002), while there was no significant difference in A20 expression between B-ALL CR patients and healthy individuals (*P* = 0.107) (Figure 1a). Interestingly, the A20 expression level in B-ALL samples was relatively different with approximately 50% of the B-ALL patients showing a relatively high A20 expression level, while the remaining 50% showed slight upregulation or a similar expression level as the healthy controls. We next examined whether the high expression level was related to disease status. We compared the expression level of A20 in newly diagnosed, untreated (de novo) B-ALL patients (median 12.252) and those with refractory/relapse B-ALL (median 21.342) (*P* = 0.616) and found no significant difference. Moreover, not only was a relatively high A20 expression level found in approximately 50% of the cases with refractory/relapse B-ALL, it was also found in 30% of cases with newly diagnosed B-ALL (Figure 1b). Therefore, it appears that the A20 expression level may not be related to disease status. This result is similar to a finding in pancreatic cancer reported by Wang et al. who showed that low A20 expression is significantly associated with pancreatic cancer behavior, but it is not the sole determinant of pancreatic cancer progression; A20 expression was not significantly associated with tumor grade, gender, age or TNM stage [26]. We further analyzed the association between the expression level of A20 and the percentage of B-ALL blasts in peripheral blood (Table 2). However, the percentage of B-ALL blasts was relatively variable in different samples, and they did not appear to be significantly associated with the blast percentage and A20 expression level in each sample (*r* = −0.023, *P* = 0.922). Thus, it would be interesting to detect A20 protein expression in each blast cell. And it is about 50% of B-ALL patients with lower white blood cells (WBC) counts compared to the rest of the patients in the same group, so we further analyzed the correlation between WBC counts and A20 expression levels to investigate whether the WBC count variation might have an impact on the difference of A20 expression detected in the B-ALL patients (Additional file 1: Table S1). However, the WBC counts did not shown significantly associated with the A20 expression levels in each group (*P* > 0.05).

**Table 2:** Clinical data of the B-ALL patients

No.	Clinical stage	Sex	Age	WBC (×10^9^/L)	Blast cells (%)	Hb	Platelets (×10^9^/L)
C1	De novo	M	31	3.49	94	111	10
C2	De novo	M	71	7.99	17.3	83	118
C3	De novo	M	28	41.9	91.9	104	15
C4	De novo	M	31	7.68	71	110	28
C5	De novo	F	16	62.3	95	76	24
C6	De novo	M	16	144.7	63	68	76
C7	De novo	F	72	17.44	82	93	20
C8	De novo	F	18	33.92	60	58	4
C9	De novo	F	17	2.5	59	106	29
C10	De novo	M	38	29.54	82	53	41
C11	De novo	F	20	4.5	86	57	8
C12	De novo	M	26	27.5	88	49	24
C13	Refractory/relapse	M	26	13.3	41	113	69
C14	Refractory/relapse	F	16	18.68	33.8	98	93
C15	Refractory/relapse	F	47	10.57	82.5	86	15
C16	Refractory/relapse	M	40	4.75	41.5	79	74
C17	Refractory/relapse	F	31	11.2	3.5	105	110
C18	Refractory/relapse	F	30	61.6	54.5	108	197
C19	Refractory/relapse	M	17	3.84	57	121	39
C20	Refractory/relapse	F	59	2.48	18	81	28

*C1 to C20* B-ALL case 1 to 20, *M* male, F female, *WBC* white blood cell, *Hb* hematoglobin.

It is known that A20 is a ubiquitin-editing enzyme with several functions. A20 was initially described as an inhibitor of TNF-induced cell death [34], and subsequent studies have demonstrated that A20 overexpression inhibits NF-κB activation in response to different stimuli [8, 35, 36]. Stable overexpression of A20 in a number of cell lines, such as human breast carcinoma MCF7 cells and murine fibrosarcoma WEHI164 cells, was shown to result in partial resistance to TNF-induced apoptosis [14, 32]. It should be noted that A20-mediated apoptosis inhibition has not been observed in all of the cell lines studied. For example, A20 overexpression in human cervical carcinoma HeLa cells, lung epithelial A549 cells, and human hepatoma HepG2 cells had no effect on apoptosis induced by the Fas receptor, lymphokine-activated killer cells, serum depletion, or oxidative stress [14, 32]. Moreover, A20 deletions and mutations are frequent in lymphoma, and its function as a crucial tumor suppressor and its deletion is closely associated with lymphoma [37]. The reason why some cell lines are protected by A20 but others are not remains unclear. Unlike a finding in T-ALL in which significantly lower A20 expression was identified [23], we found overexpression of A20 in B-ALL and its reduced expression in B-ALL CR. Thus, it appears that the roles of A20 are different in B-ALL in which it may be an inhibitor of apoptosis rather than tumor suppressor. A similar finding was reported by Frenzel et al. who showed neither mutations nor aberrant DNA methylation for A20 in 55 cases with CLL and concluded that CLL malignant development differs from most other B-cell malignancies, which show frequent A20 inactivation [34]. However, the function of A20 needs to be further investigated in B-ALL.

**Figure 2:**
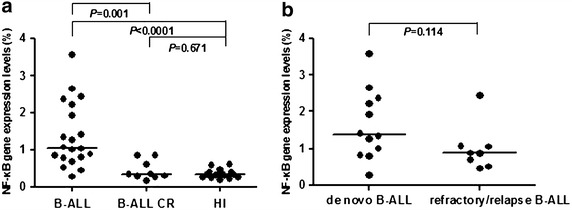
NF-κB1 expression in peripheral blood mononuclear cells from patients with B-ALL and healthy individuals. **a** Comparison of the expression level of NF-κB1 in the B-ALL, B-ALL CR and healthy individual (HI) groups; **b** comparison of the expression level of NF-κB1 in the de novo B-ALL and refractory/relapse B-ALL groups.

We also analyzed the expression level of NF-κB1, and a significantly higher expression level was found in patients with B-ALL (median 1.062) compared with healthy individuals (median 0.335) (*P* < 0.0001), while the NF-κB1 expression level was downregulated in B-ALL CR patients (median 0.339), which was significantly lower than that in the B-ALL group (*P* = 0.001) but similar to that of healthy individuals (*P* = 0.671) (Figure 2a). Although the NF-κB1 expression level appeared to be slightly high in de novo B-ALL patients (median 1.337) compared with those in the refractory/relapse B-ALL group (median 0.875), the difference was not significant (*P* = 0.114) (Figure 2b). In addition, the NF-κB1 expression level in both groups was significantly higher than that of healthy controls (*P* = 0.0003 and *P* < 0.0001, respectively) and B-ALL CR patients (*P* = 0.003, *P* = 0.008). Higher NF-κB1 levels are characteristic of cell activation and is common in cancer cells; thus, our findings are consistent with previous results [23, 34]. In fact, this finding is relatively supported by Wang et al. who demonstrated that A20 is positively correlated with the tumorigenesis of bladder polypoid disorders [21].

**Figure 3:**
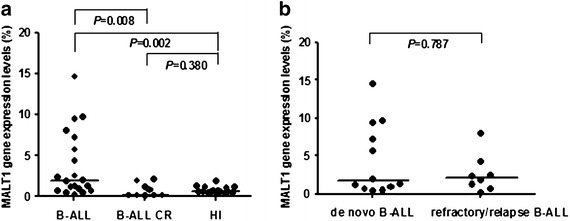
MALT1 expression in peripheral blood mononuclear cells from patients with B-ALL and healthy individuals. **a** Comparison of the expression level of MALT1 in the B-ALL, B-ALL CR and healthy individual (HI) groups; **b** comparison the expression level of MALT1 in the de novo B-ALL and refractory/relapse B-ALL groups.

We further analyzed the expression characteristics of MALT1, which positively regulated NF-κB1 as expected. MALT1 overexpression was found in the B-ALL group (median 1.938), and it was significantly higher than that in the healthy (median 0.677) (*P* = 0.002) and B-ALL CR groups (median 0.153) (*P* = 0.008), but its expression in healthy individuals and B-ALL CR patients had no significant difference (*P* = 0.380) (Figure 3a). Interestingly, similar to the A20 expression profile, the MALT1 expression level in B-ALL samples was relatively different, particularly in de novo B-ALL patients (median 1.684), and it appeared to be slightly upregulated in comparison with those in the refractory/relapse B-ALL group (median 2.096); however, the difference was not significant (*P* = 0.787) (Figure 3b).

MALT1 has proteolytic activity and controls lymphocyte activation by regulating NF-κB pathways, and it mediates rapid proteolytic cleavage and A20 inactivation [38]. Pathological alterations in the MALT1 pathway were identified in several subtypes of B-cell lymphomas, such as activated B-cell-like diffuse large B-cell lymphoma (ABC-DLBCL) [37]. In this study, MALT1 overexpression was suggested to mediate abnormal B-ALL cell activation. Unlike in T-ALL where we found significantly lower A20 expression with higher MALT1, suggesting that MALT1 might mediate A20 downregulation [23], in this study, we found that both MALT1 and A20 were overexpressed. Combined with the NF-κB1 results, these data suggested that A20 might play an oncogenic function in B-ALL, which was similar to its role in several cancers, such as glioblastomas, breast cancer, hepatocellular carcinoma, and nasopharyngeal carcinoma (NPC) [16, 18–21].

**Figure 4:**
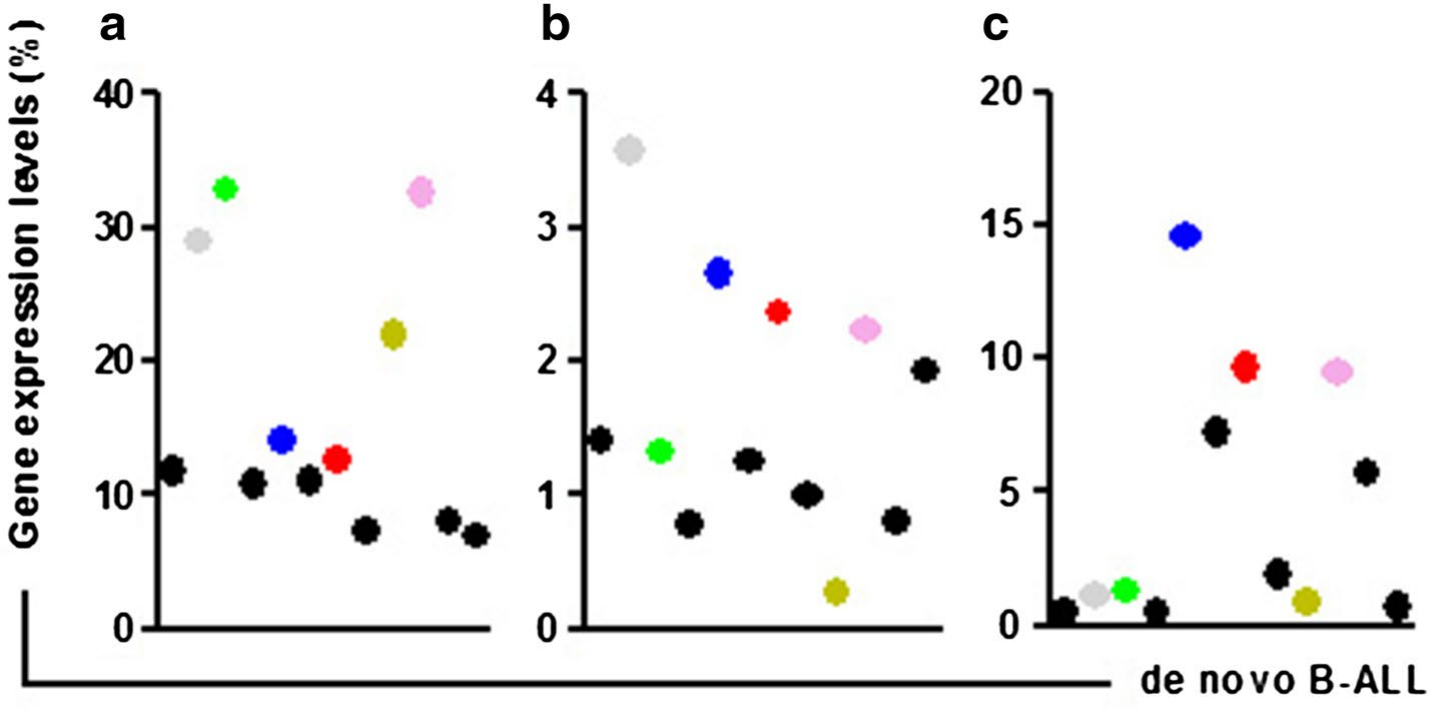
The association between the A20, NF-κB1 and MALT1 expression levels in de novo B-ALL samples. **a** A20 gene expression level; **b** NF-κB1 gene expression level; **c** MALT1 gene expression level. *Grey dots*: C2, *green dots*: C3, *blue dots*: C5, *red dots*: C7, *olive dots*: C9, *pink dots*: C10.

Overall, we demonstrated that significant overexpression of MALT1, A20 and NF-κB1 in B-ALL samples may be related to the abnormal proliferation of malignant B cells. Moreover, we found that the correlations of the expression levels of the three genes were lost in B-ALL samples because the A20 expression level is negatively correlated with that of MALT1 (*r* = −0.8601, *P* < 0.0001) and NF-κB1 (*r* = −0.9059, *P* < 0.0001), and the expression levels of MALT1 and NF-κB1 (*r* = 0.7786, *P* = 0.0004) are positively correlated in healthy individuals [23]. However, we also found a higher expression level for A20 accompanied by a higher expression level for MALT and/or NF-κB1, particularly in de novo B-ALL samples (Figure 4). This observation led us to ask whether there are two B-ALL subtypes based on the MALT1-A20-NF-κB expression pattern, and such abnormal expression characteristics may be considered biomarkers for target factors in B-ALL.

Like some epitopes on the lymphoma surface have been identified as potential targets for monoclonal antibodies [39], overexpressed genes may be considered potentially attractive targets for the development of B-ALL therapies. In the clinical, it is well known that NF-κB is a target for multiple myeloma therapy via proteasome inhibitors such as bortezomib [40]. Moreover, there are two types of small molecule inhibitors for MALT1 in preclinical studies. One inhibitor is the phenothiazine derivative mepazine, which has been shown to have promising anticancer properties in B cell lymphoma subtypes [41]. The other inhibitor is MI-2, which directly binds to MALT1, and irreversibly suppresses its protease function and exhibits selective activity against ABC-DLBCL cell lines in vitro and ABC-DLBCL xenotransplant tumors in vivo. It may be worth investigating the anti B-ALL effects of such MALT1 inhibitors [42]. For A20 target inhibition, siRNA was used to downregulate A20, and greatly inhibiting A20 expression slowed tumor cell growth in culture and mice, and it was also shown to induce apoptosis in glioblastomas [19]. Moreover, current renewed appreciation of the unique molecular signatures of tumors holds promise for personalized chemotherapeutic regimens, hopefully comprising specific MALT1-A20-NF-κB pathway-targeting agents [43].

## Conclusions

Recent reports have indicated that A20 expression is increased in a number of solid human tumors [43]. In this study, we first demonstrated the overexpression and loss of the normal expression pattern of MALT1, A20, and NF-κB1 at the molecular level, indicating that their manner of regulation in B-ALL may be more complex. The altered MALT1-A20-NF-κB pathway may contribute to the pathogenesis of B-ALL, and this pathway may be considered a potentially attractive target for the development of B-ALL therapeutics. However, these findings are based on results from a limited case analysis, and further study involving more samples is needed to determine representative results.

## Additional file

Additional file 1:
**Table S1.** The correlation between A20 expression levels and WBC counts.
