# Supplementary material for: Overexpression of MALT1-A20-NF-κB in adult B-cell acute lymphoblastic leukemia
Source: Cancer Cell Int. 2015 Jul 25;15:73. doi: 10.1186/s12935-015-0222-0 (PMC4514975; doi:10.1186/s12935-015-0222-0)
Supplement: Additional file 1: — Table S1. The correlation between A20 expression levels and WBC counts. [file 12935_2015_222_MOESM1_ESM.doc]

**Additional table S1** The correlation between A20 expression levels and WBC counts

| NO. | Clinical stage | WBC (×10^9^/L) | A20 expression levels | Correlation* |
| --- | --- | --- | --- | --- |
| C1 | de novo | 3.49 | 11.78 | *P*=0.784 |
| C2 | de novo | 7.99 | 29.02 |
| C3 | de novo | 41.9 | 32.99 |
| C4 | de novo | 7.68 | 10.84 |
| C5 | de novo | 62.3 | 14.26 |
| C6 | de novo | 144.7 | 11.23 |
| C7 | de novo | 17.44 | 12.72 |
| C8 | de novo | 33.92 | 7.48 |
| C9 | de novo | 2.5 | 21.99 |
| C10 | de novo | 29.54 | 32.53 |
| C11 | de novo | 4.5 | 8.19 |
| C12 | de novo | 27.5 | 7.15 |
| C13 | refractory/relapse | 13.3 | 9.91 | *P*=0.138 |
| C14 | refractory/relapse | 18.68 | 11.66 |
| C15 | refractory/relapse | 10.57 | 23.41 |
| C16 | refractory/relapse | 4.75 | 25.88 |
| C17 | refractory/relapse | 11.2 | 19.28 |
| C18 | refractory/relapse | 6.16 | 23.49 |
| C19 | refractory/relapse | 3.84 | 27.64 |
| C20 | refractory/relapse | 2.48 | 8.48 |
| C21 | complete remission | 5.37 | 3.76 | *P*=0.211 |
| C22 | complete remission | 3.22 | 18.95 |
| C23 | complete remission | 1.68 | 18.49 |
| C24 | complete remission | 1.53 | 8.54 |
| C25 | complete remission | 4.82 | 2.65 |
| C26 | complete remission | 5.27 | 3.25 |
| C27 | complete remission | 3.42 | 2.23 |
| C28 | complete remission | 5.68 | 3.01 |
| C29 | complete remission | 4.51 | 7.11 |
| N1 | healthy individuals | 6.99 | 11.11 | *P*=0.787 |
| N2 | healthy individuals | 9.53 | 8.84 |
| N3 | healthy individuals | 9.20 | 12.16 |
| N4 | healthy individuals | 8.28 | 8.14 |
| N5 | healthy individuals | 6.35 | 5.22 |
| N6 | healthy individuals | 7.40 | 3.69 |
| N7 | healthy individuals | 6.73 | 10.44 |
| N8 | healthy individuals | 5.68 | 6.77 |
| N9 | healthy individuals | 7.12 | 6.14 |
| N10 | healthy individuals | 9.52 | 8.11 |
| N11 | healthy individuals | 7.56 | 12.29 |
| N12 | healthy individuals | 7.31 | 8.63 |
| N13 | healthy individuals | 4.37 | 11.03 |
| N14 | healthy individuals | 8.18 | 8.66 |
| N15 | healthy individuals | 5.47 | 11.48 |
| N16 | healthy individuals | 4.80 | 9.57 |

* Spearman’s correlation.
